# A Chemical Ionization Residual Gas Analyzer for the Sensitive Detection of Atmospheric Trace Gases

**DOI:** 10.1021/jasms.6c00116

**Published:** 2026-06-18

**Authors:** Kevin A. Wokosin, Matthew C. Zoerb, Timothy H. Bertram, Steven J. Kregel

**Affiliations:** † Department of Chemistry, 5228 University of Wisconsin-Madison, Madison, Wisconsin 53706, United States; ‡ Department of Chemistry and Biochemistry, 7173 California Polytechnic State University, San Luis Obispo, California 93407, United States; § Mund-Lagowski Department of Chemistry and Biochemistry, 6496 Bradley University, Peoria, Illinois 61625, United States

## Abstract

We have developed a compact, chemical ionization mass spectrometer for the sensitive detection of atmospheric trace gases using a commercial residual gas analyzer (RGA). A high-pressure ion molecule reactor interface incorporating two radiofrequency (RF)-only octupoles, powered by Wisconsin Oscillator RF power supplies, is used to focus ions through three stages of pumping before analysis via a modified RGA. We operated this instrument with benzene cation cluster reagent ions generated using a ^210^Po α emitter to enable the efficient ionization, transfer, and detection of analyte ions and ion–molecule clusters. Using single ion monitoring, the instrument demonstrated high sensitivity to dimethyl-1,1,1-*d*_3_ sulfide, corresponding to a 20 s 3σ limit of detection (LOD) of 18 ppt. This detection threshold is limited by the magnitude and variability in the background determination. We characterized the impact of tuning instrument pressures and ion optics on sensitivity and ion clustering to demonstrate the potential for enhanced sensitivities and electronically controllable clustering modalities. The application of the instrument to the measurement of ambient trace gases, in scan mode, was tested by sampling continuously through a window inlet on the fourth floor of the UW-Madison chemistry building (Madison, WI) for 3 days during the summer of 2025, resulting in the detection of four unique, time-correlated ammonia clusters via peak integration. The simple, robust design and relatively low cost of the instrument highlight the utility of commercial residual gas analyzers as mass analyzers for gas-phase measurements of trace species.

## Graphical abstract

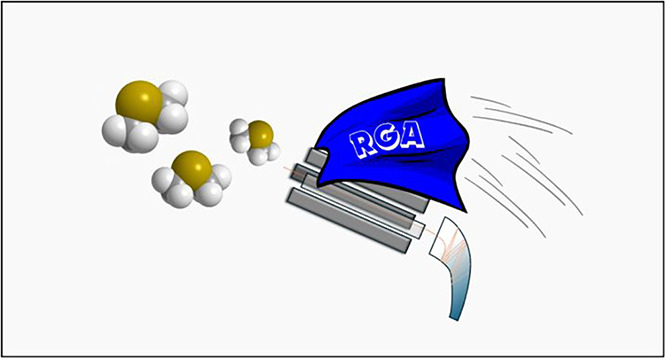

## 1 Introduction

Over the past 2 decades, chemical ionization mass spectrometry (CIMS) has emerged as a powerful tool in atmospheric chemistry research for the sensitive, selective, and rapid time response measurement of a wide array of trace gases.^1,2^ These instruments play a central role in indoor and outdoor field research, where they have been used to quantify the sources of reactive gases and to track the multigenerational photooxidation of these molecules to constrain atmospheric chemical mechanisms.^3–5^ These same CIMS instruments have also become a cornerstone of laboratory investigations of atmospheric processes such as the formation and growth of secondary organic aerosols and the chemical cycling of oxidants central to tropospheric ozone formation.^6–9^

Existing CIMS instrumentation used within the atmospheric chemistry community includes custom-designed instruments utilizing commercial quadrupole mass analyzers (e.g., Extrel and Thermo Fisher MSQ) coupled with a wide array of chemical ionization reactors and high-throughput ion transmission elements. Complete, commercially available quadrupole-based CIMS instruments (Q-CIMSs) have also been developed (e.g., Ionicon, THS Instruments).^10^ Both commercial and custom Q-CIMS instruments featured prominently in atmospheric chemistry research prior to 2010 and continue to be used for selected applications. The development of the atmospheric pressure ionization time-of-flight (API-ToF) mass spectrometer, based on the compact, field-deployable ToF designed by TofWerk, led to the rapid emergence of both custom-built and commercially available ToF-CIMS instruments.^11–13^ Existing ToF-CIMS instruments utilize a wide array of reagent-ion chemistries, permitting the measurement of most reactive chemical species known in the atmosphere, either directly or through chemical transformation.^4,13–21^ Further, these instruments have achieved remarkably high detection sensitivities, vanishingly small background count rates, and have been interfaced with chromatographic and ion mobility techniques to connect measured ions confidently with atmospheric molecules, enhancing their selectivity when sampling complex mixtures.^22,23^ Since 2010, ToF-CIMS instruments have been deployed to all corners of Earth, including high-altitude research aircraft, ocean-going research vessels, and both the most remote and most populated cities.^24–26^

However, the success of commercial ToF-CIMS instruments has displaced a prior cornerstone in atmospheric chemistry: the development of custom instrumentation and the training of early-career scientists in instrument development. Further, while technologically superior in almost every metric, acquisition of a commercial ToF-CIMS is unattainable by large fractions of the atmospheric chemistry community due to their purchase and maintenance costs. Currently, a void exists for accessible, lower-cost, autonomous CIMS instruments with detection limits that are suitable for field and laboratory measurements (<100 ppt in 1 min).

An instrument meeting these requirements could: (1) reshape routine monitoring of atmospheric trace-gas composition, (2) enable rapid emergency response deployments (e.g., spatiotemporal measurements of hazardous materials following environmental release), and (3) reduce economic barriers to chemical instrumentation. In this paper, we describe a CIMS instrument for directly sampling atmospheric trace gases built using a commercially available residual gas analyzer (RGA), inspired by Ng et al.’s Aerosol Chemical Speciation Monitor (ACSM) and Saltzman et al.’s “mini-CIMS” RGA for continuous measurements of dissolved gases in seawater.^27,28^ The proof-of-concept RGA-based CIMS described in this manuscript represents a critical step toward the future goals of an open-source, lower-cost CIMS instrument for sensitive trace gas detection built around an affordable, commercially available mass analyzer platform.

The cornerstone of this instrument is the open-source Wisconsin Oscillator we developed in 2021.^29,30^ The Wisconsin Oscillator enables us to easily operate multiple radiofrequency (RF) ion guides and ensure efficient ion transmission from the high-pressure ionization region (*P* > 50 Torr) to the low-pressure region (*P* < 5 × 10^–6^ Torr) housing the modified residual gas analyzer.

## 2 Experimental Section

### 2.1 Instrument Description

We have developed a modular, accessible, field-deployable mass spectrometer with multiple differentially pumped vacuum stages, low-cost ion guides, and a residual gas analyzer (RGA)-based mass analyzer. The instrument hardware can be built from all-new parts for <50k USD and potentially for much less if buying used or employing existing components. This modular design enables the coupling of a higher-pressure ion source with an RGA and a small quadrupole mass filter-based mass analyzer, which requires much lower pressures to operate. This system is designed for flexibility and modification to facilitate the attachment of a variety of different ion sources while employing the same transfer optics and mass analyzers.

The existing instrument is mounted into a custom-built extruded aluminum frame (61 × 91 × 91 cm^3^) for ease of use and flexibility for instrument development modifications. The instrument core (Figure 1) is contained within 48 × 38 × 66 cm^3^ volume, permitting it to be packaged in a much smaller enclosure and deployed to a wide range of sampling platforms, especially if operated with peripherals (e.g., power supplies and backing pumps) connected with a long umbilical cord.

**Figure 1:**
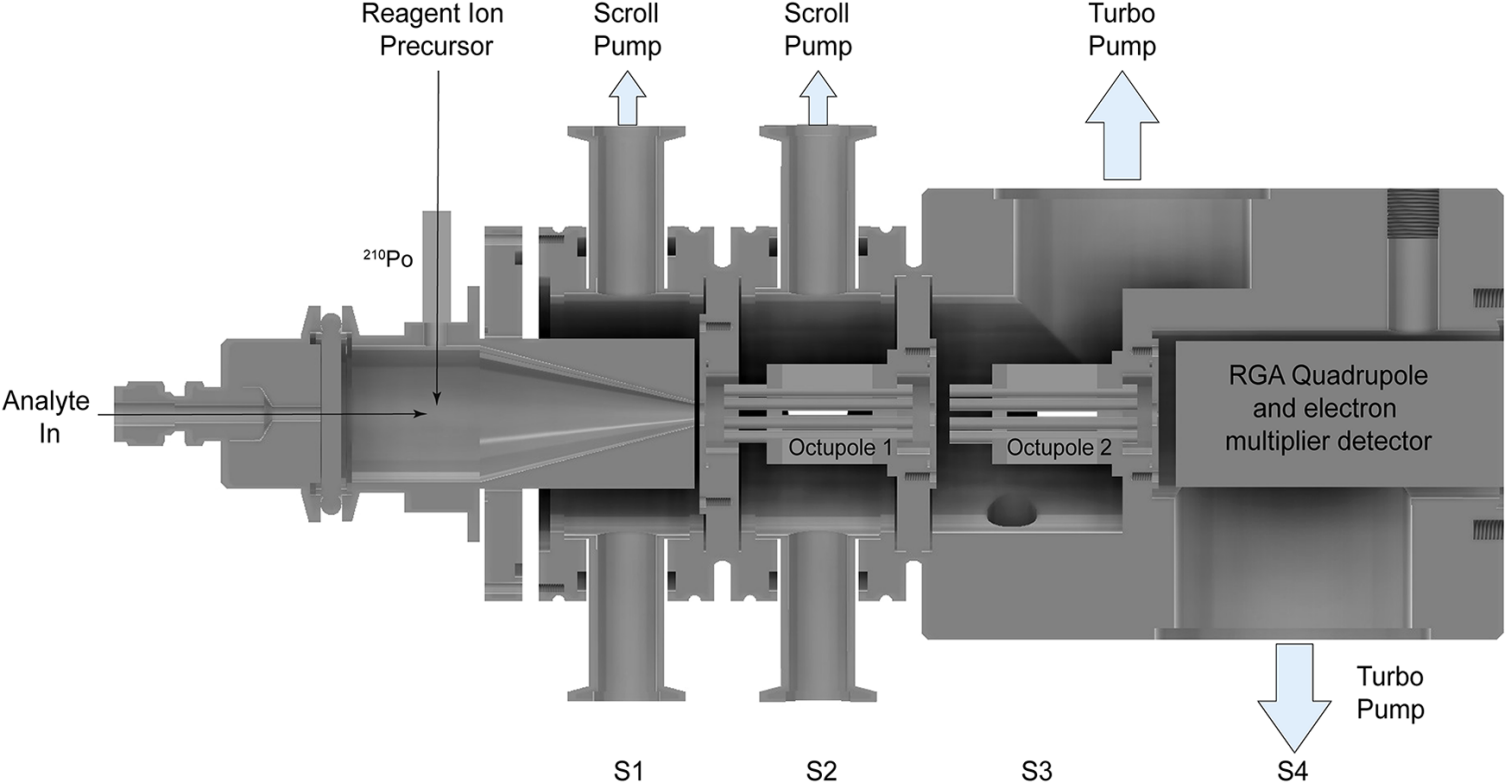
Schematic of the instrument coupled to a chemical ionization source and four stage (S1–S4) differentially pumped interface coupled to the RGA quadrupole. The high-pressure interface consists of: (i) ion–molecule reaction (IMR) chamber (S1, 70 Torr), (ii) collisional dissociation chamber (CDC) (S2, 3 Torr), (iii) stage 3 that houses a second octupole ion guide (S3, 1.5 × 10^–2^ Torr), and stage 4 containing the RGA’s quadrupole and electron multiplier detector (S4, 2× 10^–6^ Torr).

As shown in Figure 1, the instrument core consists of four differentially pumped vacuum chambers, which contain the ion source, ion transfer optics, and the residual gas analyzer. For this work, we have configured our instrument for chemical ionization using flow through a commercial ^210^Po α emitter (NRD P-2021-1000) to generate benzene cluster cation reagent ions. The use of a ^210^Po α emitter requires appropriate permitting and safety infrastructure measures. The ion molecule reactor (IMR) is based on established designs from the atmospheric chemistry community originating from Huey’s research group.^31–34^ Two transfer octupoles enable efficient ion transmission from the high-pressure chemical ionization source through the differentially pumped stages to a final chamber containing the mass analyzer, a Pfieffer PrismaPlus RGA with the filament removed and extractor cage modified to enable the injection of externally generated ions. Direct current (DC) voltages to power the ion optics are supplied by a commercial multichannel power supply (Ardara Technologies) and RF voltages are applied to the octupole ion guides using open-source Wisconsin Oscillators.^29,30^ A custom electronics box containing a National Instruments DAQ (NI9205) was constructed to monitor the pressure in each stage of the mass spectrometer and digitally control the output voltages from the multichannel DC power supply. Additionally, the DAQ controls a set of three MKS mass flow controllers (10, 5,000, and 20,000 sccm) located within the custom electronics box. These mass flow controllers provide gas flow to the reagent-ion source and generate the standardized gas mixtures used for routine sensitivity calibrations. A custom LabView program interfaces with the DAQ and the RGA to control all instrument operations, acquire mass spectra, and continually record the pressure in each chamber.

Because the chemical ionization ion source involves gas flows on the order of 3 L min^–1^ into the IMR during operation, a dedicated scroll pump is required to meet the pumping needs of the ion source. The IMR chamber (S1) is pumped by a 110 L min^–1^ dry scroll pump (Varian SH-110), which is typically throttled down to achieve a sampling pressure of 70 Torr; however, diagnostic experiments have been run in the pressure range of 30–100 Torr. The second pumping stage (S2) is pumped to ∼3 Torr via a throttled 170 L min^–1^ dry scroll pump, either an Agilent IDP10 or Ebara PDV-500 500 L min^–1^ dry vacuum pump. This second scroll pump also serves as a backing pump for the two 80 L s^–1^ turbo pumps (Agilent TwisTorr 84 FS), which pump the second octupole (S3) to 1.5 × 10^–2^ Torr and the RGA (S4) chamber to ∼1 × 10^–6^ Torr during operation. The pinhole diameters between each pressure stage are as follows: 0.381 mm (S1/S2), 0.518 mm (S2/S3), and 1.27 mm (S3/S4).

### 2.2 Reagent-Ion Generation

The reagent-ion precursor (benzene gas) is generated by diluting a certified compressed gas standard (200 ppm benzene in N_2_, 2% analytical uncertainty, Airgas) with ultrahigh purity (UHP) N_2_ generated from liquid nitrogen boil off to achieve a benzene mixing ratio of 10 ppm using mass flow controllers (±10%). The total reagent flow rate into the IMR is controlled by an in-line critical orifice (1.3 L min^–1^) which subsamples the diluted reagent-ion precursor and serves to keep the permeable Teflon tubing at positive pressure relative to the atmosphere, preventing the diffusion of contaminants into the ion generation lines. Due to the inherent safety hazards posed by benzene, the exhaust stream from the pumps must be connected to a properly vented exhaust manifold or exhausted through an activated charcoal or other air scrubber device for safe operation of the instrument. Downstream of the orifice, gas flow is passed through an α-emitting polonium-210 source (NRD P-2021-1000 at 10 mCi initial activity). N_2_^+^ ions are formed via electron transfer reactions with α particles and subsequently ionize neutral benzene molecules. These benzene cations then cluster spontaneously with neutral benzene via attractive, noncovalent interactions to form a distribution of benzene cluster cations depending on the pressures and benzene neutral mixing ratio (see Section 3.3).^35,36^ The IMR is typically held at 70 Torr, where residence times of the analyte molecules are estimated to be on the order of 100 ms, significantly longer than the time scale for most weakly bound clusters (e.g., water or benzene) to reach equilibrium. Kim et al. studied the parameters controlling the benzene cation cluster distribution (C_6_H_6_)_*n*_·(C_6_H_6_)^+^ and determined that the reagent ion within the ion–molecule reaction chamber was primarily in the form of the benzene dimer or larger clusters for operational conditions like those used in this instrument.^18^ This conclusion is supported by studies showing that the dissociation energy of the benzene cation dimer is significantly higher than that of the trimer or larger benzene cation clusters, suggesting that chemical ionization by benzene cluster cations in our IMR configuration proceeds primarily through clusters that are at least the size of the benzene cation dimer.^37^

Previous work with benzene cation chemical ionization mass spectrometry demonstrated that select volatile organic compounds (VOCs) including dimethyl sulfide (DMS), isoprene, terpenoids, and aromatic compounds can be ionized by benzene cation clusters.^17,18,38^ Within the IMR, analytes are ionized via interactions with benzene cation cluster reagent ions (denoted as (C_6_H_6_)*_n_* ·(C_6_H_6_)^+^) with the cluster distribution and analyte ionization energy (IE) both playing significant roles in the observed ion chemistry. More recently, Puttu et al. introduced a thermodynamics-based framework to classify analytes into three categories (low, mid, and high) based on their ionization energy.^19^ Analytes with IE smaller than 8.69 eV (low IE class) primarily undergo charge transfer with both reagent ions (Reaction 1); analytes with IE between 8.69 and 9.24 eV (mid IE class) undergo charge transfer with (C_6_H_6_)^+^ (Reaction 1, where *n* = 0) and potential benzene adduct formation (Reactions 2 and 3); analytes with IE larger than 9.24 eV (high IE class) could undergo adduct formation, proton transfer, or hydride abstraction (Reactions 2–5).^19^ The ligand exchange product (Reaction 3) was previously reported for isoprene, dimethyl sulfide, and select alkenes; however, the reaction pathway is not known.^17,18^

R1 $${\left(C_{6}H_{6}\right)}_{n}\cdot {\left(C_{6}H_{6}\right)}^{+}+M\to M^{+}+{\left(C_{6}H_{6}\right)}_{n}$$

R2 $$C_{6}H_{6}^{+}+M⇋{C_{6}H_{6}\cdot M}^{+}$$

R3 $${\left(C_{6}H_{6}\right)}_{n}\cdot {\left(C_{6}H_{6}\right)}^{+}+M\to M^{+}\cdot \left(C_{6}H_{6}\right)+{\left(C_{6}H_{6}\right)}_{n-1}$$

R4 $$C_{6}H_{6}^{+}+M\to {MH}^{+}+C_{6}H_{5}^{•}$$

R5 $$C_{6}H_{6}^{+}+M\to {\left[M-H\right]}^{+}+C_{6}H_{7}^{•}$$

All the ions in our system undergo several energetic collisions as they are transported from the IMR to the RGA for mass analysis. These collisions are energetic enough to alter the observed (C_6_H_6_)*_n_* ·(C_6_H_6_)^+^ distribution, and thus, we are unable to accurately measure the benzene cluster cation distribution present within the IMR. The relative abundance of ions observed by the detector is controlled by the ion optic voltages and the pressure of the IMR/CDC regions, indicating that our choice of instrument parameters influences the observed ion chemistry within the IMR and the survival rate of weakly bound ionic clusters through the instrument. Two operational set points were utilized in this work: high declustering (higher voltage gradient, higher transmission) and low declustering (lower voltage gradient and transmission) depending on the experimental objectives (see Table 1 and Figure 2).

**Figure 2:**
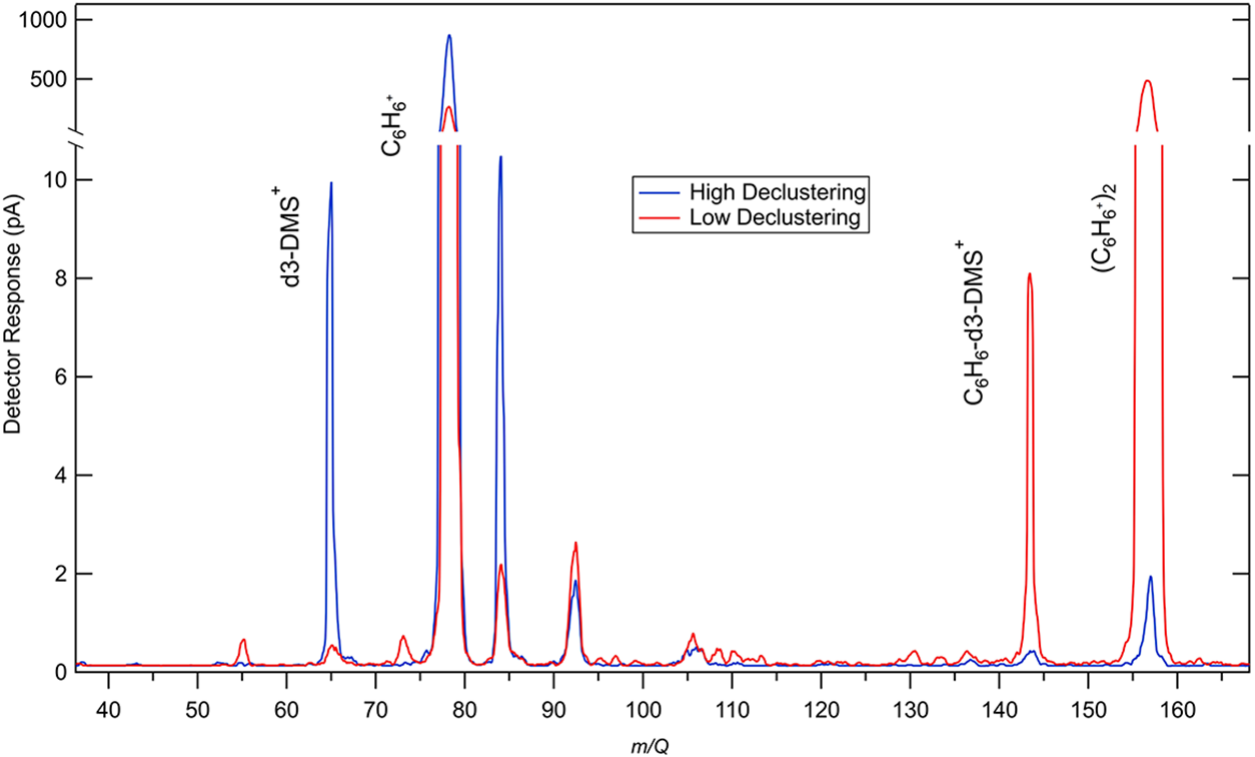
Mass spectrum acquired in high (blue) and low (red) declustering operational modes with benzene diluted in N_2_ to a neutral concentration of 10 ppm and DMS-*d*_3_ diluted in ultra-zero air to a concentration of 10 ppb for high and low-declustering conditions. The peaks at mass to charge (*m*/*Q*) 84 and 92 *m*/*Q* represent trace contaminants from the benzene cylinder as they are also observed while sampling zero air.

**Table 1:** Ion Optics Configuration for High and Low-Declustering Instrument Modes with Instrument Pressures of 70 Torr (IMR) and 4 Torr (CDC)

Mode	Ion current (pA) (C_6_H_6_)^+^/(C_6_H_6_)_2_^+^	IMR (V)	P1 (V)	O1 (V)	P2 (V)	O2 (V)	P3 (V)
High-declustering	975/1.93	260	240	157	121	114	108
Low-declustering	307/545	132	130	127	123	123	109

### 2.3 Ion Molecule Reactor and Ion Transfer Optics

Once generated, the benzene cluster cations react with sampled air within the ion molecule reactor (IMR). The first section of the stainless-steel IMR chamber is 2.5 cm long and has an inner diameter (ID) of 3.5 cm (Figure 1). The second section of the IMR chamber is 5 cm in length and is linearly tapered to reduce the ID to 0.6 cm at the exit. Ambient air is sampled into the front of the IMR through a 0.5 mm critical orifice at a rate of 1.7 L min^–1^. Reagent ions are orthogonally injected into the IMR ∼ 2 cm downstream from the atmospheric sampling inlet. Based on the pumping speed and volume of the IMR chamber, we estimate a residence (and reaction) time of ∼100 ms for the ions within the IMR. This residence time can be altered by changing the pressure and/or pumping speed. The IMR flow is subsampled, through a second critical orifice (500 μm) biased to a more negative voltage than the IMR, into the collisional dissociation chamber (CDC), which contains a short octupole ion guide powered by the Wisconsin Oscillator (4.76 cm, *r*_0_ = 6.35 mm, 1.6 MHz, 115 V_p–p_) for focusing and cooling the expansive ion beam.^29,30^ Following the ion guide in the CDC region, the ions pass through another biased aperture to a second identical octupole in the S3 chamber, before passing through the third and final biased aperture and being injected into the RGA mass analyzer (Figure 1).

For this work, we utilized two sets of instrument parameters (Table 1), a high-declustering mode which shifted the observed reagent and product ions toward molecular ions with an observed benzene monomer to dimer ratio of 505:1, and a low-declustering mode with softer ionization, mostly formed adduct ions and an observed benzene monomer to dimer ratio of 4:7 (Table 1).

### 2.4 Residual Gas Analyzer

The RGA employed for this instrument is a PrismaPlus QMG 220 system with a compact secondary electron multiplier (C-SEM) option from Pfeiffer Vacuum. This model offers Ethernet connectivity, a small form factor, and a low power draw. It has a mass range of 1–200 atomic mass units (amu) and achieves unit mass resolution. We coupled the PrismaPlus to our custom CIMS source by removing the OEM electron impact ion source and grid to enable the injection of externally generated ions into the quadrupole mass filter. For all of the work presented here, the applied voltage on the compact electron multiplier within the RGA was 2100 V.

The RGA operates in two primary sampling modes: the scan mode and multiple ion detection (MID). Scan mode cycles through a defined mass range at a user-determined rate and saves the intensity of the detector signal into a mass spectrum. Multiple ion detection (MID) cycles through a list of user-defined mass windows (typically 1 Th wide) to create a time series of intensity for a list of mass-to-charge (*m*/*Q*) targets. The dwell time and duty cycle for each *m*/*Q* of interest can be set to anywhere from fractions of a second up to a few minutes, depending on the goals of the experiment.

Due to the intended operating conditions, the PrismaPlus RGA quadrupole assembly is designed with a fixed bias of ∼+100 V relative to ground to mitigate electrons from the OEM ion source reaching the detector. We have removed the OEM ion source, but are unable to change the RGA float bias, creating an inherent limitation in the ion optics voltage gradient. Given this restriction, our upstream ion optics and CIMS ion source must be biased positively with respect to the RGA quadrupole to facilitate efficient ion injection into the mass analyzer.

## 3 Results and Discussion

### 3.1 Sensitivity and Absolute Accuracy

To determine the suitability of our instrument for atmospheric analysis, we assessed its sensitivity to dimethyl-1,1,1-*d*_3_ sulfide (DMS-*d*_3_). Previous studies have studied DMS using benzene cluster cation chemistry, and this scheme enables us to assess the performance of our instrument in the context of prior measurements. Atmospherically relevant concentrations of DMS-*d*_3_ (0–5 ppbv) were produced by mixing 4–6 L min^–1^ UHP zero air with 1–10 cm^3^ min^–1^ DMS-*d*_3_ (Airgas, 1.14 ppm in N_2_, 5% analytical uncertainty) and overflowing the inlet. The absolute accuracy of this technique is driven primarily by uncertainty in the calibration standard gas cylinder. In the high-declustering mode, the instrument response to DMS-*d*_3_ was measured with single ion monitoring at 20 s averaging time of the M^+^ peak at *m*/*Q* 65. A time series for the calibration experiment is shown in Figure 3a demonstrating the time response of the instrument. The detector response (in picoamperes (pA)) was observed to be a linear function of the delivered DMS-*d*_3_ up to approximately 1 ppbv with a slope of 1.18 pA ppbv^–1^ (Figure 3b). The dependence of the sensitivity on absolute humidity was not assessed, but has been discussed for previously reported systems utilizing benzene cluster cation CIMS with identical ion–molecule reactor geometries and operating conditions.^18,38^

**Figure 3:**
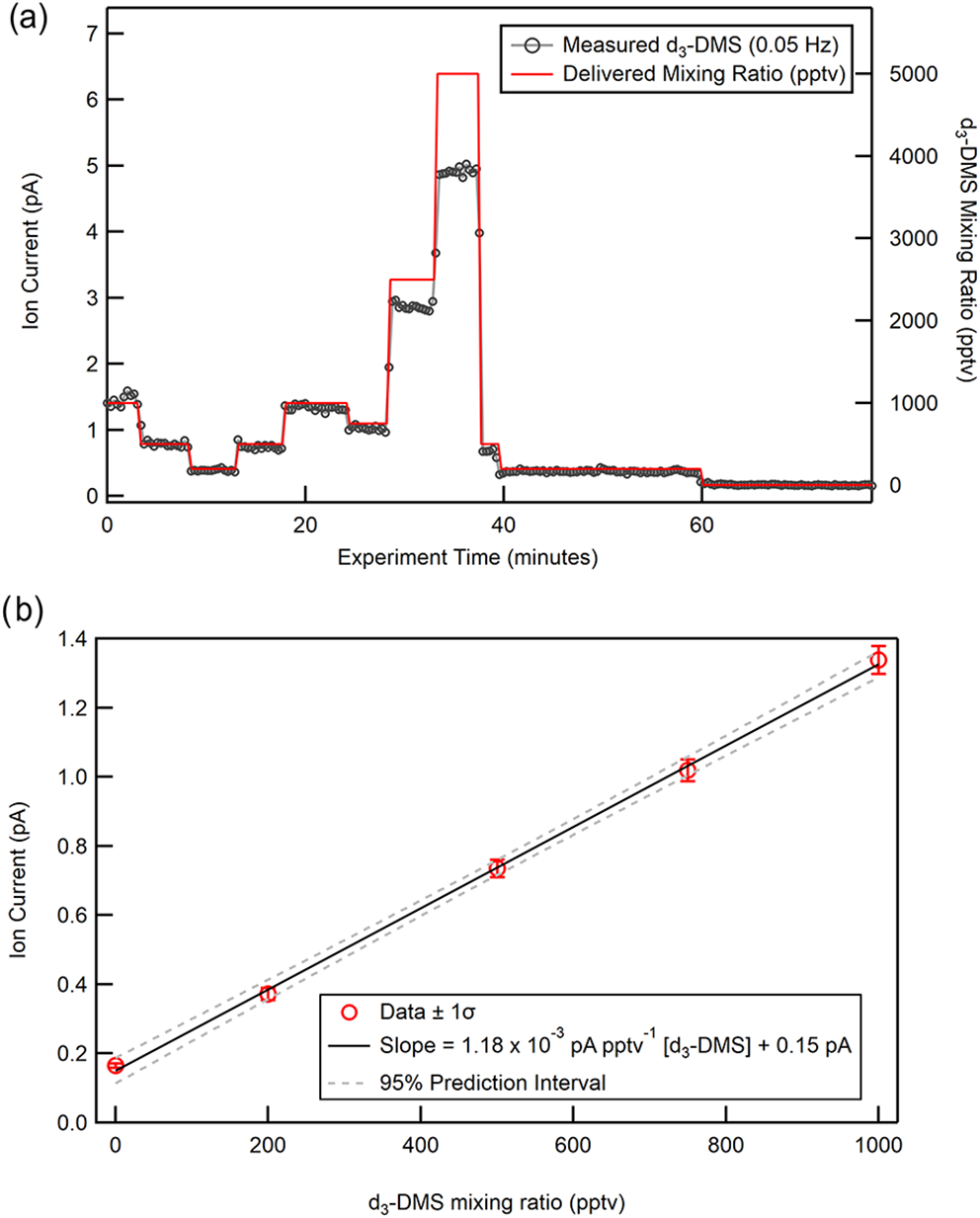
(a) Time series for DMS-*d*_3_ mixing ratio and instrument response during calibration experiment in high-declustering mode monitoring exclusively 65 *m*/*Q* with 20 s averaging time and operational pressures of 70 Torr IMR and 4 Torr CDC. Instrument responses at mixing ratios >1 ppbv are lower than expected from the linear trend. (b) Calibration curve for DMS^+^-*d*_3_ (65 *m*/*Q*).

In the low-declustering mode, the instrument response to 1.14 ppbv of DMS-*d*_3_ was assessed with 20 s signal averaging at both M^+^ and the M^+^ benzene adduct, giving single-point sensitivities of 0.27 ± 0.02 and 0.64 ± 0.04 pA ppbv^–1^, respectively, with the same instrument pressures as the high-declustering calibration (IMR 70 Torr, CDC 4 Torr). We note that the sensitivity of the instrument is dependent on the activity of the ^210^Po source, which decays with a half-life of 138 days. Measurements reported here were made with a ^210^Po source that was approximately 90 days old, so we expect higher sensitivity with a newer ^210^Po source.

### 3.2 Limit of Detection

The detection limit of the instrument is a strong function of the variance in the instrument background. The instrument response while sampling UHP zero air in the high-declustering mode was determined to be 0.16 ± 0.01 pA (20 s signal averaging) at the nominal mass of the DMS-*d*_3_ molecular ion (D_3_CSCH_3_; 65 *m*/*Q*) due to the noise inherent to the RGA detector electronics and signal processing (Figure 4). For comparison, the instrument response to 200 pptv DMS-*d*_3_ (also shown in Figure 4) is 0.37 ± 0.02 pA (20 s signal averaging), indicating that the detection limit is well below the lowest measured calibrant concentration of 200 pptv DMS-*d*_3_. Given the standard deviation of the background zero (6.9 × 10^–3^ pA), and the slope of the calibration curve from the high-declustering mode (1.18 pA ppbv^–1^), we calculate a 20 s 3σ LOD of 18 ppt and a 10σ LOQ of 58 ppt for DMS-*d*_3_.

**Figure 4:**
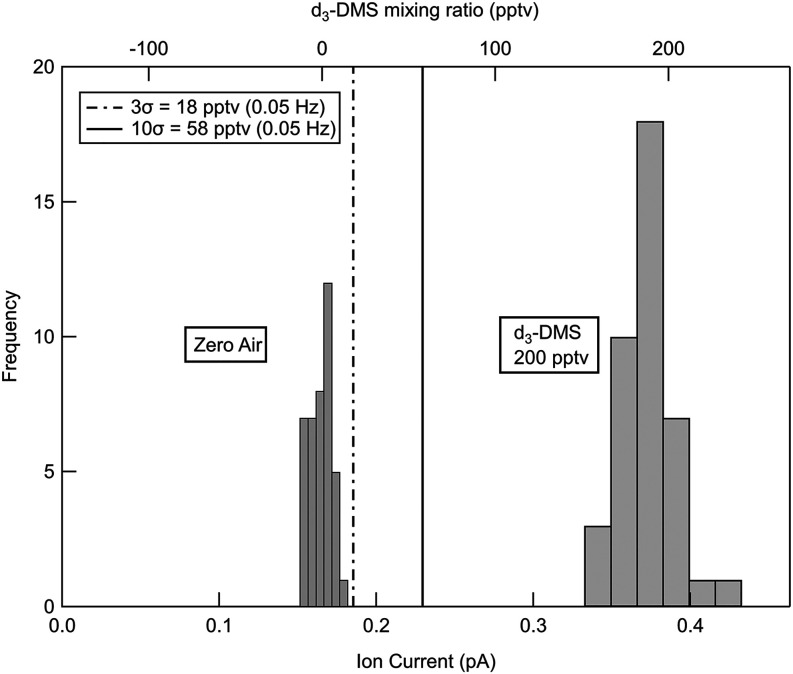
Histogram of the measured DMS-*d*_3_ background and a constant concentration of 200 pptv from the high-declustering calibration curve experiment. The 20 s 3σ LOD and 10σ LOQ for DMS-*d*_3_ are included for context and plotted on the top axis.

### 3.3 Optimization of Instrument Pressures

The dependence of the instrument sensitivity on the IMR and CDC pressures was assessed in the low-declustering operational mode, and these tests were performed with an Ebara PDV-500 500 L min^–1^ dry vacuum pump backing the turbo pumps. The IMR pressure was varied between 60 and 200 Torr while the CDC was held constant at 4 Torr. As expected, the sensitivity to the DMS-*d*_3_ molecular ion and the DMS-*d*_3_–benzene adduct (C_6_H_6_^+^·D_3_CSCH_3_; 143 *m*/*Q*) increased with increasing IMR pressure, primarily due to increased analyte number density as shown in Figure 5.

**Figure 5:**
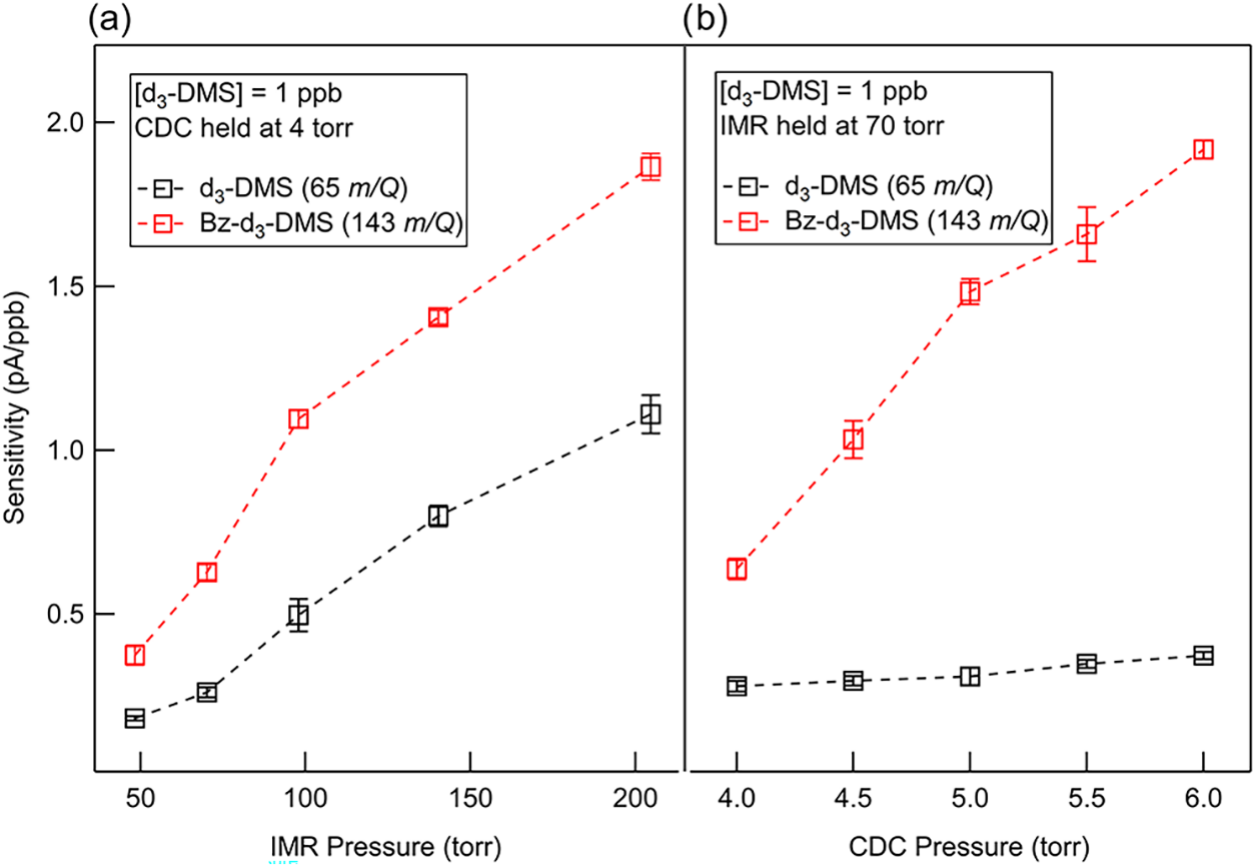
DMS sensitivity (pA ppb^–1^) vs IMR pressure (a) and CDC pressure (b) for the DMS-*d*_3_ molecular ion (65 *m*/*Q*) and DMS-*d*_3_–benzene adduct (143 *m*/*Q*) in the soft-declustering operational mode.

The response of the benzene (C_6_H_6_^+^; 78 *m*/*Q*) and benzene dimer cluster ((C_6_H_6_)·(C_6_H_6_)^+^; 156 *m*/*Q*) reagent ions to IMR pressure increased up to ∼90 Torr then fell back down, possibly due to higher order clusters outside of the mass range of the RGA taking a larger fraction of the reagent ions or increased charge transfer to contaminants with lower ionization energies than benzene such as toluene.

In a separate experiment, the CDC pressure was varied between 4 and 6 Torr, while the IMR was held constant at 70 Torr. Increased CDC pressure resulted in an increased signal response for the benzene dimer cation and the DMS-*d*_3_–benzene adduct, with smaller increases for the benzene and DMS-*d*_3_ molecular ions (Figure 5). We expect the reduced collisional energy at higher CDC pressures to promote an increased production/survival of the adduct species over the molecular ion product.

Given that the calibration curve reported in Figure 3 was collected for an IMR pressure of 70 Torr and CDC pressure of 4 Torr (these parameters were selected based on similarity to previously published work on C_6_H_6_ CIMS to enable more accurate comparisons with well-characterized ion chemistry), improvements in sensitivity are possible over the reported calibration curve. For example, running the instrument with CDC up to 6 Torr and IMR up to 200 Torr could yield significant improvements in sensitivity. However, higher-pressure conditions will result in different secondary chemistry and are different from the results reported in existing benzene cluster CIMS literature.^17–19,38^ Nevertheless, using these increased sensitivities in conjunction with our measured zero value produces an estimated 20 s 3σ LOD of <10 ppt.

## 4 Atmospheric and Laboratory Observations

To demonstrate the capability of the instrument for ambient atmospheric measurements, ambient air was sampled continuously from the fourth floor of the UW-Madison Chemistry building in Madison, Wisconsin, for 3 days (May 30 to June 2, 2025). The inlet flow rate was controlled by the pull of the instrument in the standard configuration (1.7 L min^–1^). Mass scans were collected with a dwell of 2 s amu^–1^ over the range of 50–200 amu, resulting in a mass spectrum approximately every 5 min. Low-declustering ion optics were employed to encourage larger benzene cluster ionization chemistry and adduct formation for the detection of atmospheric trace gases.

A time series for our data was generated using the batch peak fitting tools in Igor Pro 9 to fit the 857 scans collected fully autonomously over the 3-day sampling period. The reagent-ion peaks (C_6_H_6_^+^ and (C_6_H_6_)·(C_6_H_6_)^+^) showed good mass stability and consistent intensity throughout the sampling period with a characteristic peak width of ∼0.7 *m*/*Q*, in line with peak shapes from calibration scans. Peak integration for less abundant ion signals (<3 pA) was complicated by interferences and artifacts such as spikes in the detector current with a characteristic peak width too narrow to be representative of a true ion signal. To remove spike interferences, integration results with a peak width <0.2 *m*/*Q* were filtered out after peak fitting integration for ion signals with maximum values <3 pA.

Ammonia (NH_3_) is a known atmospheric trace gas in south-central Wisconsin. During summer in Madison, WI, tropospheric ammonia emissions stem from fertilizer application, livestock excrement, and combustion emissions.^39^ Prior reported tropospheric NH_3_ mixing ratios range from 0.1 to hundreds of ppbv depending on emission sources, boundary layer dynamics, and the acidity of aerosol particles present in the atmosphere.^40^ During this time, Madison’s air quality experienced impacts from transported wildfire smoke.

Previous benzene cluster CIMS calibrations for NH_3_ report humidity-dependent sensitivity to the benzene ammonia adduct, with increasing sensitivity as relative humidity increases.^19^ Due to the softer-declustering ion optics in our instrument and the presence of water vapor in ambient air, we expect to observe the benzene ammonia adduct as well as additional hydrated clusters. In our ambient measurements, we observed four unique, time-correlated ion clusters consistent with hydrated ammonia–benzene adducts: NH_3_·(H_2_O)·(H_3_O)^+^ (54 *m*/*Q*), NH_3_·C_6_H_6_^+^ (95 *m*/*Q*), (C_6_H_6_)·(NH_3_)·(H_2_O)^+^ (113 *m*/*Q*), and (C_6_H_6_)·(NH_3_)·(H_2_O)_2_^+^ (131 *m*/*Q*), with the dominant ammonia species detected as the (C_6_H_6_)·(NH_3_)·(H_2_O)^+^ (113 *m*/*Q*) cluster (Figure 6). We elect not to report NH_3_ mixing ratios, as the instrument inlet manifold was not optimized for NH_3_ measurements, and we lacked a robust calibration system for characterizing the instrument response during the observation period. Nonetheless, the uncalibrated ion intensities reported in Figure 6 highlight the instrument’s response to atmospheric NH_3_ fluctuations over a wide dynamic range.

**Figure 6:**
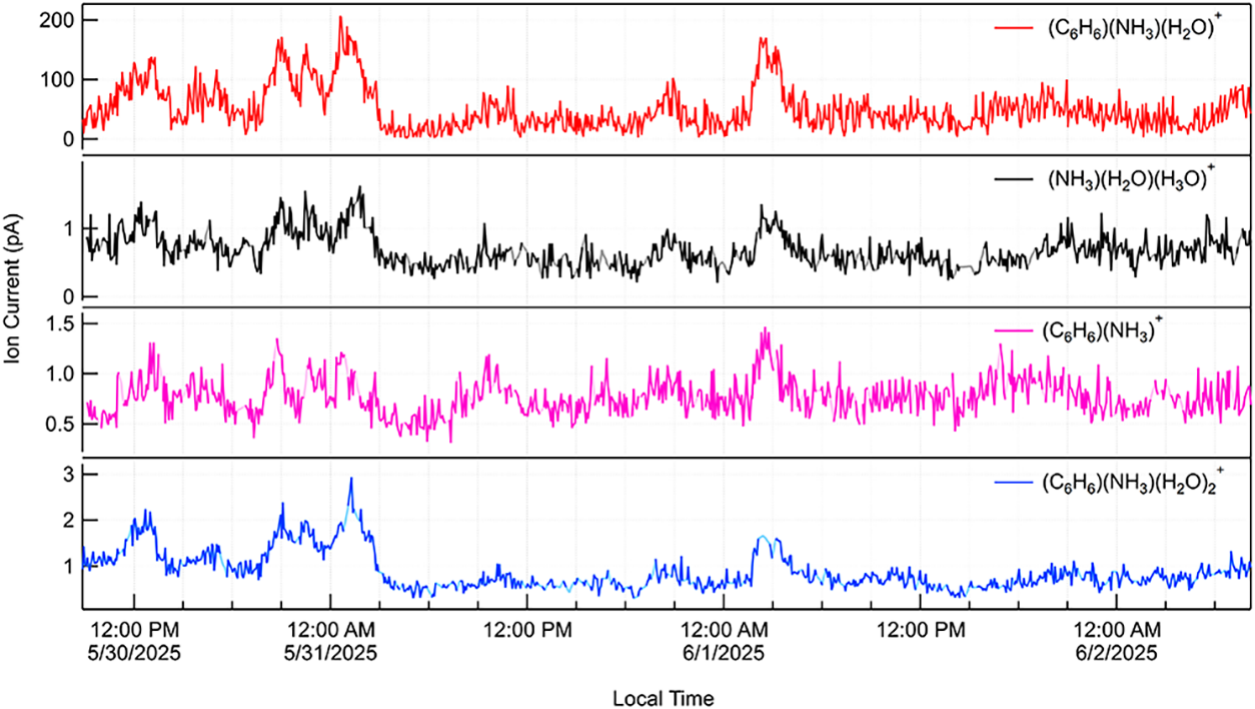
Time series of four unique ion clusters consistent with formulas of (NH_3_)·(H_2_O)·(H_3_O)^+^, (C_6_H_6_)·(NH_3_)^+^, (C_6_H_6_)·(NH_3_)·(H_2_O)^+^, and (C_6_H_6_)·(NH_3_)·(H_2_O)_2_^+^ detected in ambient air in Madison, WI. Peak fitting for each species was performed on 857 scans. Smaller intensity peaks (<3 pA) were subject to artifacts, which resulted in erroneous peak fits with widths too narrow to represent a true ion signal. Peak areas from integrations with a width <0.2 have been filtered out after peak fitting. A linear interpolation is plotted in a lighter shade trace to aid visualization of these data.

## 5 Conclusions and Future Directions

Here, we demonstrate that commercial residual gas analyzers can be used as the mass analyzer for chemical ionization mass spectrometry with DMS-*d*_3_ sensitivities of >1 pA ppbv^–1^ and a 20 s 3σ LOD of 18 ppt, enabling gas-phase measurements of trace species. In comparison to existing instruments described in the literature, this represents a significant advance in reducing the size, complexity, and cost of making ambient VOC measurements. The system described in this manuscript was constructed as a proof-of-concept to demonstrate that an RGA can be used as the mass analyzer for atmospheric trace gas measurements. Moving forward, we look to improve on the first generation of this instrument as we work toward the goal of an open-source CIMS instrument. This first-generation instrument was not built with the intention of replication, but to demonstrate that RGA mass analyzers, when combined with high efficiency ion generation and transmission, are capable of atmospheric trace gas detection. Having passed this milestone, we are now working on scalable hardware and software solutions that are reproducible outside of our laboratories. In what follows, we highlight specific improvements we will target as we construct this next-generation instrument.

The most obvious near-term improvement is to use a newer RGA system. Newer RGA models have mass ranges of up to 320 amu and can provide a direct connection to the output of the electron multiplier, enabling the highly sensitive ion counting methods of traditional quadrupole CIMS instruments. In the longer term, we envision modifications to the Wisconsin Oscillator circuit that will enable it to directly power a mass-resolving quadrupole. Tests of modified Wisconsin oscillator circuits on the bench have produced sinusoidal waveforms in excess of 1 kV_p–p_ at 1.5 MHz, which is sufficient for driving a small quadrupole such as those found within RGAs. Such an advancement would enable the low-cost integration of a home-built quadrupole mass filter and ion detection system, reduce the voltages needed for upstream ion optics, and enable monitoring of both positive and negative ions—greatly increasing the utility of RGA-based CIMS instruments.

In addition to upgrading the mass analyzer, we anticipate that sensitivity gains can be achieved through optimization of ion transport between the ion source and the analyzer. The relatively high pressure of the S2 and S3 stages can hinder the forward motion of ions through multipolar ion guides, but incorporating ion funnels into either or both stages would enable control of the ions’ forward motion and better constrain the ions radially to improve overall ion transmission.

The natural decay of the ^210^Po source necessitates frequent in-field calibrations, and the radioactivity of the source exhibits regulatory difficulties for transportation. An ion source that generates a variety of reagent ions using photoionization or a clean electrical discharge would eliminate many of these logistical considerations and provide a high-stability reagent-ion signal over long time periods.^15,41^

Though fully realizing these improvements will take some time, we hope that they will—in conjunction with the work presented here—reinvigorate the tradition of custom instrumentation within the atmospheric chemistry community and expand access to highly capable instrumentation.
